# Multivariate filter methods for feature selection with the $\varvec{\gamma }$-metric

**DOI:** 10.1186/s12874-024-02426-9

**Published:** 2024-12-19

**Authors:** Nicolas Ngo, Pierre Michel, Roch Giorgi

**Affiliations:** 1 Aix Marseille Univ, Inserm, IRD, SESSTIM, Sciences Économiques & Sociales de la Santé & Traitement de l’Information Médicale, ISSPAM, Marseille, France; 2 Aix Marseille Univ, CNRS, AMSE, Aix-Marseille School of Economics, Marseille, France; 3 Aix Marseille Univ, APHM, Inserm, IRD, SESSTIM, ISSPAM, Hop Timone, BioSTIC, Marseille, France

**Keywords:** Atrial fibrillation, Classification, Feature selection, $\gamma$-metric

## Abstract

**Background:**

The $\gamma$-metric value is generally used as the importance score of a feature (or a set of features) in a classification context. This study aimed to go further by creating a new methodology for multivariate feature selection for classification, whereby the $\gamma$-metric is associated with a specific search direction (and therefore a specific stopping criterion). As three search directions are used, we effectively created three distinct methods.

**Methods:**

We assessed the performance of our new methodology through a simulation study, comparing them against more conventional methods. Classification performance indicators, number of selected features, stability and execution time were used to evaluate the performance of the methods. We also evaluated how well the proposed methodology selected relevant features for the detection of atrial fibrillation, which is a cardiac arrhythmia.

**Results:**

We found that in the simulation study as well as the detection of AF task, our methods were able to select informative features and maintain a good level of predictive performance; however in a case of strong correlation and large datasets, the $\gamma$-metric based methods were less efficient to exclude non-informative features.

**Conclusions:**

Results highlighted a good combination of both the forward search direction and the $\gamma$-metric as an evaluation function. However, using the backward search direction, the feature selection algorithm could fall into a local optima and can be improved.

## Introduction

Adding features to classification models is not always beneficial to the problem at hand. In fact, some features may degrade the model’s predictive performance. This study aims to propose a new methodology for selecting relevant features in classification problems. The motivation for this work stems from the need to improve the detection of atrial fibrillation (AF) from electrocardiogram (ECG) data. AF is the most common cardiac arrhythmia, characterized by an irregular cardiac rhythm and an often rapid heart rate. Its prevalence increases with age [1], and it is associated with a significantly higher risk of stroke - up to five times greater [2, 3].

AF is typically detected through a 24-hour ECG recording, which is performed in a hospital or specialized facility. If confirmed, patients can be prescribed anticoagulants to reduce the risk of blood clot formation, and subsequently, the risk of stroke. However, AF is often asymptomatic and can be intermittent, with episodes lasting from a few minutes to a few days before the heart returns to normal sinus rhythm (NSR), sometimes for longer periods than 24–48 hours. This irregularity makes it challenging to detect AF unless the patient is continuously monitored with ECG.

Emerging technologies, particularly mobile health (mHealth), are being developed to monitor patients non-invasively and continuously, facilitating long-term data collection for suspected AF cases [4–6]. These tools allow for the collection of physiological data, accurately reflecting cardiac activity. From this data, various features (e.g., the average time between two consecutive heartbeats) can be derived and used as inputs for predictive classification models to detect abnormal heart activity.

However, in real-world data analysis, it is often unclear whether each extracted feature is relevant for classifying heart rhythms (i.e., NSR vs AF). As the dimensionality of a dataset increases (i.e., the number of features grows), the risk of including irrelevant or redundant features also increases. These extraneous features can reduce the predictive capacity of classification models. Feature selection addresses this issue by reducing the dataset’s dimensionality, which can enhance model performance, reduce computation time, and simplify model complexity. Moreover, by eliminating irrelevant features, the resulting models are better equipped to generalize to new data, avoiding overfitting. In turn, such a model can facilitate the interpretation of results by researchers, clinicians, and decision-makers.

Feature selection is a statistical process that can be applied to both regression and classification models. In this study, we focused on distinguishing AF from NSR using Holter-based ECG data and applied feature selection in the context of supervised classification models. Feature selection methods are commonly grouped into three categories [7]. The first is ‘filter methods’; these methods are applied before model construction. They evaluate the importance of individual features (or set of features) based on predefined metrics. The second category is ‘wrapper methods’; these methods rely on a classification model to assess features and by evaluating subsets of features using model performance criteria. The last category is ‘embedded methods’; In these methods, feature selection is integrated into model-building process. The feature selection process can be univariate, where each feature is evaluated independently and assigned an importance score. However, univariate methods do not account for potential correlations between features. To address this, multivariate methods evaluate subsets of features, considering the relationships among them.

In 2017, a new metric called the $\gamma$-metric was proposed by Pons et al. [8] to evaluate the discriminatory power of numeric features in classification tasks. The authors demonstrated that features with the highest $\gamma$-metric values yielded the highest accuracy in univariate logistic regression models for AF detection. In a more recent study, [9] examined the performance of the $\gamma$-metric as a univariate filter for feature selection, comparing it with three other univariate filters in the context of AF detection. The results showed that $\gamma$-metric produced comparable results to existing ranking methods, though it was univariate and only considered the relevance of individual features.

In the present study, we developed a new methodology for multivariate filter feature selection in classification, using the $\gamma$-metric as an evaluation function. The $\gamma$-metric is associated with a specific search direction (and therefore a specific stopping criterion). As three search directions are used, we effectively created three distinct methods. We assess the performance of these methods through a simulation study and compare them to seven conventional feature selection methods. Finally, we applied all ten methods (the three novel and the seven conventional) to the detection of AF using ECG data.

The paper is organized as follows: Methods section explains the computation of the $\gamma$-metric and its integration into three new feature selection methods, along with a description of the conventional feature selection methods. Simulation study section details the simulation study used to validate the three novel methods and compares their performance with existing methods. Application section presents the application of these methods to AF detection and their related results. Finally, Discussions section concludes the paper with a discussion of the simulation results and potential perspectives for using the $\gamma$-metric in classification models.

## Methods

To select the best subset of features in a classification model, one approach is to evaluate all possible combinations of candidate features and then choose the subset that optimizes an evaluation function. However, for a dataset with *p* features, this requires evaluating $2^p - 1$ combinations, which can be extremely time-consuming and computationally expensive [10]. For example, with only 20 features, over a million combinations must be evaluated. Therefore, attempting to explore the entire feature space is impractical. Instead, more efficient strategies should be employed to explore the feature space in a way that ensures a reasonable solution can be found without evaluating every possible feature combination. Various feature selection methods have been developed based on different mathematical concepts and exploration strategies, but the objective remains the same: to identify the best subset of relevant features as closely as possible [11]. Feature space exploration generally follows four key steps [10]: (i) Defining a subset generation procedure, often referred to as search direction, (ii) selecting an evaluation function to assess feature relevance (in this study, the $\gamma$-metric is used), (iii) establishing a stopping criterion, and (iv) validating the feature selection process, (i.e., validating steps (i), (ii), and (iii)).

In step (i), the subset generation procedure defines how the feature space will be explored, specifically how features are added or removed from candidate subsets.This process, known as the search direction, determines how features are selected for evaluation. For example, in an exhaustive search, all possible subsets of features are evaluated.

Step (ii) involves the evaluation function, which measures the relevance of the feature (or subset of features) generated in the previous step. This function determines how important each feature or subset is for the model in question.

Step (iii) defines the stopping criterion, which indicates when the feature selection process should halt. Two methods with identical generation procedures and evaluation functions can yield different results if they employ different stopping criterion.

Finally, step (iv) is the validation of the feature selection process itself. This occurs outside the selection process and ensures that the chosen subset of features is indeed relevant and performs well in the selected model.

In the following sections, we describe the $\gamma$-metric, which is used as the evaluation function in step (ii). We then present three proposed multivariate filter feature selection methods, each employing a different search direction (step (i)) and using the $\gamma$-metric as an evaluation function (step (ii)) with a strict stopping criterion (step (iii)). Finally, we compare the performance of these methods with more conventional feature selection methods through a simulation study and by applying them to the task of discriminating between AF and NSR using Holter-based ECG data (step (iv)).

### $\varvec{\gamma }$-metric as an evaluation function

When developing the $\gamma$-metric for classification tasks, Pons et al. [8] main idea was to represent the *K* classes by *p*-dimensional ellipsoids, with *p* being the number of features. Each ellipsoid is characterised by the position of its center and the length and direction of its axes. The $\gamma$-metric computes the distance between the centers of each ellipsoid by taking into account the overlap of the ellipsoids. If an overlap exists, then the $\gamma$-metric will be negative; otherwise, it will be positive. In order to compute the $\gamma$-metric, we consider a set of *n* observations $\{\varvec{X}_{i}\}_{i = 1,...,n}$ with $\varvec{X}_{i} \in \mathbb {R}^p$. These observations belong to one of the *K* classes, i.e., $Y_i = 1,...,K \ \forall i = 1,...,n$. The first step is to group all observations from each class *k* and compute the associated class covariance matrix:

1 $$\begin{aligned} \varvec{W}_{k, p} = \text {Cov}(\varvec{X}_{i}|\varvec{Y}_{i} = k), \end{aligned}$$

$\varvec{W}_{k, p}$ is a diagonalizable, symmetrical positive semi-definite $p \times p$ matrix, with eigenvalues $\{ \lambda _{k, j} \}_{j = 1,...,p}$, and eigenvectors $\{ u_{k, j} \}_{j = 1,...,p}$ representing, respectively, the length and direction of the *p* axes of the ellipsoid. This ellipsoid is centered at $\varvec{\mu }_{k}$, the mean vector of observations for class *k*. Hence, each class is represented by an ellipsoid, and the $\gamma$-metric represents the sum of the distances between each pair of ellipsoids. More specifically, for each pair of ellipsoids, the $\gamma$-metric represents the distance between the centroids minus the distance between the centroids and the borders of each ellipsoid; this ensures that any overlap of the ellipsoid is taken into account. For $K = 2$, there are only two ellipsoids, and the $\gamma$-metric is the distance between the two centroids minus the centroid-border distance of each. For $K> 2$, the distance between all pairs of ellipsoids is computed and then summed. More details on the computation of the $\gamma$-metric can be found in Appendix A.

In Eq. 1, the covariance matrix can be estimated either with the empirical estimator or the shrinkage estimator, depending on the ratio between features and observations. In reality, in the context of a large-scale dataset (i.e., $p \gg n$) the empirical covariance matrix is a poor estimation of the covariance matrix, as it can lead to an ever-increasing number of null eigenvalues and to singularity of the covariance matrix [12]. This could pose a problem for the computation of the $\gamma$-metric, since the eigenvalues are used in the computation of the distances involved (see Appendix A for details on the computation of the $\gamma$-metric). Therefore, for the present study, we used the shrinkage estimation [13] of the covariance matrix when the number of observations for at least one class was smaller than the number of features.

### Multivariate feature selection method using the $\varvec{\gamma }$-metric

We used the $\gamma$-metric as an evaluation function to develop a novel multivariate feature selection methodology. Among all the subsets of candidate features, we looked for the one which maximized the $\gamma$-metric value. Specifically, we used three search directions (see below). Each search direction used a specific means to generate and explore the feature space. Therefore, each was separately associated with the $\gamma$-metric as an evaluation function. The three corresponding algorithms had the same strict stopping criterion. Hence there were three distinct feature selection methods using the $\gamma$-metric as an evaluation function. All three are described below:

Backward search (GAMMA_BACK). For this method, the value of the $\gamma$-metric is first computed for the whole set of features. Next, the $\gamma$-metric value of all the possible subsets with one less feature is evaluated. If the value of the $\gamma$-metric of any given subset is strictly greater than that of the first candidate subset, then the former subset is retained; otherwise the algorithm stops and returns the first candidate subset. This process is repeated with the highest value being compared to the $\gamma$-metric value of the subset retained in the previous step. If the $\gamma$-metric value is strictly greater, the iterations continue either until there are no more features to remove or the $\gamma$-metric value cannot be increased by removing a feature of the candidate subset.

Forward search (GAMMA_FORW). In this method, the value of the $\gamma$-metric is first computed for each feature individually; the first candidate subset will be the feature with the highest value. Next, the $\gamma$-metric of all the possible subsets with one more feature is evaluated. If the $\gamma$-metric value of the given subset is strictly greater than that of the first candidate subset, then the former subset is retained; if not, the algorithm stops and returns the first set. The same process is then repeated: the $\gamma$-metric value of all the possible subsets with one more feature is evaluated; then the highest value is compared to the $\gamma$-metric value of the subset retained in the previous step. If the $\gamma$-metric value is strictly greater, the iterations continue either until there are no more features to add or the $\gamma$-metric value cannot be improved by adding a feature to the candidate subset.

Best first search (GAMMA_BF). With this method, the value of the $\gamma$-metric is first computed for each feature; the first candidate subset is formed by the feature with the highest $\gamma$-metric value. Next, the $\gamma$-metric of all the possible subsets with one more feature is evaluated. If the $\gamma$-metric value of a given subset is strictly greater than that of the first candidate subset, then the former subset is retained; if not the algorithm returns the first set. In the next step, the $\gamma$-metric value of all possible neighbour subsets, with one more feature, is evaluated and the highest value is then compared to the $\gamma$-metric value of the subset retained in the previous step. If the $\gamma$-metric value is strictly greater, the iterations continue; if not, the candidate subset is not directly returned. The best first search provides the possibility to go back to the second-best subset of features of the previous step and to continue the iterations with these poorer candidate features. In this way, this search direction is less likely to return a local maximum for the evaluation function. The iterations continue either until there are no more features to add to the candidate subset or the $\gamma$-metric value cannot be improved or the number of ‘go back to a less optimal candidate subset’ iterations reaches a maximum.

The simulation study and healthcare application was conducted using R to compute the $\gamma$-metric. The shrinkage estimation was performed with the R package corpcor [14] and the cov.shrink function.

### Conventional feature selection methods

Several other feature selection methods have been proposed for supervised classification purposes which are more conventional in nature. In order to compare the performance of our novel $\gamma$-metric multivariate filter feature selection methods with these existing methods, we considered seven feature selection methods (4 filter methods, 2 wrapper methods and one embedded method). The general principle behind each of these methods is described below. Related technical details for some methods are provided in Appendix B.

Chi-squared filter (CHI2), this univariate filter method uses the Chi-squared statistic [15] to measure the dependence between the feature and the class. To do this, continuous features are discretized, the Chi-squared statistic is estimated, and Cramer’s V is used as a ranking score for each feature. Cramer’s V values close to 1 indicate a strong association between the feature and the class (see Appendix B: Chi-squared section).

Correlation-based feature selection (CFS) [16] is a multivariate filter feature selection method. It evaluates subsets of features on the basis of the hypothesis that “good feature subsets contain features highly correlated with the class, yet uncorrelated to each other” [16]. CFS ranks features according to an evaluation function based on correlations. This method assumes that irrelevant features have a low correlation with the class, and should be ignored. For a classification problem, CFS first discretizes numeric features and then entropy measure is used to estimate the degree of association between discrete features [16, 17]. The best-first search direction is used to generate the subsets to evaluate. The mathematical computation of the criterion used in CFS can be found in Appendix B: Correlation-based feature selection section.

Least absolute shrinkage and selection operator regression (LASSO) [18] is a multivariate embedded feature selection method. In a linear regression equation, the LASSO method adds a penalty term that discourages the model from assigning too much importance to any single feature. The penalty applied here is the L1 norm, which is the sum of the absolute values of the regression coefficients. This method allows some coefficients to be shrunk exactly to zero, effectively performing feature selection. A penalty parameter, which controls the strength of the regularization, is calibrated using a cross-validation.

Random forest importance (RFI) is a multivariate filter method. It is embedded in the random forest algorithm. More specifically, it computes the mean decrease accuracy (MDA) score for each feature in order to rank them [19]. This score describes how much accuracy the model loses by permuting values of the feature. The idea is that for each feature, its score is computed by comparing the accuracy of the full prediction with the accuracy of the prediction when the feature values are randomly permuted. A high MDA value means that the permutations of the feature greatly impacted the accuracy, hence that the feature is important for a good accuracy. A low MDA value means that the values of the feature does not impact the prediction of the model. Once the score is computed for each feature, they can be ranked by order of importance. The features with the best ranks are then selected.

Stepwise AIC selection (STEP) is a multivariate wrapper method. The idea is to compare the AIC (a measure of the goodness-of-fit and complexity of a model) of the models by removing or adding features to the model. Start with the model with no features (only the intercept) and add the feature that reduces the AIC the most. Then repeat by adding or removing the feature that reduce the most the AIC. The methods is stopped when we cannot decrease the value of the AIC by removing or adding a feature.

Symmetrical uncertainty (SU) [20] is an univariate filter method based on the entropy and the information gain. This method is a variant of the mutual information [21] where we compute the entropy of all the data and the entropy of the candidate subset of features. In the mutual information, the entropy tends to be biased toward features with a large number of different values, in the SU method a normalization of the mutual information is applied to lower this bias (see Appendix B: Symmertrical uncertainty section).

Support Vector Machine Recursive Feature Elimination (SVM-RFE) is a multivariate wrapper method [22]. The method uses a recursive feature elimination searching procedure associated with a support vector machine classification model [23, 24]. The idea is to train an SVM model with all the features at first step. In the process of building an SVM model, a weights vector $\varvec{v}$ of the features is estimated. This weights vector is a linear combination of the training sample and the weight of each feature can be used as a ranking criteria of the features. The features with the smallest values are then eliminated and we train again the SVM with the remaining features.

To apply the feature selection methods CFS, CHI2, RFI and SU we used the implementation of the R package FSelector [17]. In addition, since CHI2, RFI and SU implementations return only a ranking of the features, with regard to their importance, we used a cutoff based on the biggest difference of importance score for each method. This way, the number of features selected was not fixed beforehand. For the $\gamma$-based feature selection methods (GAMMA_BACK, GAMMA_FORW, and GAMMA_BF), the FSelector package offer the possibility to use a custom evaluation function with their implementation of the backward, forward and best first search direction functions. So we plugged in the $\gamma$-metric value function. For the LASSO feature selection method, we used the glmnet package [25, 26]. The step function from stats package was used to apply the stewise AIC feature selection method. Last the SVM-RFE method was applied with the package mlr3 [27].

## Simulation study

### Design of the simulation study and assessment of the feature selection methods’ performances

To validate each of our three feature selection methods using the $\gamma$-metric as an evaluation function, we considered three distinct scenarios of binary classification problems. In these scenarios we explored the trade-off between number of observations and the number of features; class balancing and separability; and the effect of multicollinearity. Accordingly, we incorporated informative features with a fixed non-null effect and non-informative features with a null effect. In scenario 1, to assess whether each method selected informative features (and did not select non-informative features) in classical context, we considered situations where the number of observations was much higher than the number of features, with strong positive and negative effects. We also included a feature with a much lower effect. In scenario 2, we considered (i) more features than observations, (ii) both balanced and unbalanced classes, and (iii) situations where the two classes were strongly separated or not by the features. In scenario 3, we explored the efficiency of the methods in complex contexts with various pattern of correlations between the features, we considered (i) constant and non-constant levels of correlation, and (ii) three different levels of correlation. In all scenarios, we aimed to test whether the feature selection methods can guarantee good classification performance, select all the features that are truly informative and disregard all the non-informative features.

The classification performance was assessed using three criteria: (i) the area under the curve (AUC) which is an overall indicator of the model predictive performance, (ii) the sensitivity (iii) and specificity of the model. The feature selection process for each method was assessed by computing the average number of informative and non-informative features selected. The stability of the feature selection methods was assessed using the Jaccard index [28]. Additionally, we measured the execution time of the feature selection process, in seconds, of each method.

We applied three $\gamma$-metric based feature selection methods, plus the seven conventional methods presented above. The performance of the ‘FULL’ model, which uses all available features without any selection, is included as a reference for comparison. This model serves as a baseline to illustrate the impact of feature selection on predictive performance.

In each of the three scenarios for each of the eleven methods (the three $\gamma$-metric based methods, the seven conventional methods and the FULL method which returns all available features), we generated two datasets with specific parameters: a training dataset and a validation dataset. After applying a given feature selection method, the selected features were included in a logistic regression model developed from the training dataset. Using the validation dataset, we assessed the classification performance indicators. For each scenario, we repeated the generation of the training and validation datasets (both generated with the same sample size), the feature selection step, and the assessment of the classification performance 50 times. At each repetition of the simulation, we ensured that all the feature selection methods are applied on the same data (also assessment of the classification performances was done on the same validation dataset for all models). Stability was assessed using the Jaccard index [28] with pairwise comparison of the selected features over all the repetitions.

### Data generation

To generate data, we considered the following logistic equation, with $\rho _i$ being the probability that the observation *i* is a class 1 type:

$$\begin{aligned} \rho _i = \frac{\exp (\varvec{X_i \beta })}{1 + \exp (\varvec{X_i \beta })}, \quad \forall i \in 1,...,n. \end{aligned}$$

With $\varvec{\beta }$ the vector of effects, including an intercept $\beta _0$. We considered *p* informative features associated with a non-null effect (i.e., $\beta _j \ne 0, \forall j \in \{1,...,p\}$). We also added $p'$ features having a null-effect (i.e., $\beta_j = 0, \forall j \in \{p+1,...,p+p'\}$). $\varvec{X}$ is thus the matrix of $\mathbb {R}^{n \times (p+p'+1)}$ where each column is a vector of *n* realisations of a Gaussian distribution. The class of the observation *i* is then defined by $Y_i \sim \text {Bernoulli}(\rho _i)$. The different values of $\varvec{\beta }$ for both scenario 1 and 2 are reported in Table 1 (see Appendix D for details on the choice of the $\varvec{\beta }$ values). The generation of $\varvec{X}$ described above applies for both scenario 1 and 2. For scenario 3, in order to generate data with multicollinearity, we used a multivariate Gaussian distribution with a variance-covariance matrix $\varvec{\Sigma }$. There was, in the generation process, groups of features, that were correlated together, but features from different groups were not correlated together. One group of features was generated with no correlation (within and with the other groups). All features had a variance of 1. Hence, the covariance matrix $\varvec{\Sigma }$ was a block diagonal matrix, with each block being the variance-covariance matrix, noted $\varvec{\Sigma }_g$, of a group of features. The construction of the covariance matrix was similar as the work of [29] with $\varvec{\Sigma_g}$ defined as:

$$\begin{aligned} \varvec{\Sigma }_g = \left( \begin{array}{cccc} 1 & \alpha _{ij} & \cdots & \alpha _{ij} \\ \alpha _{ij} & 1 & \cdots & \alpha _{ij} \\ \vdots & \vdots & \ddots & \vdots \\ \alpha _{ij} & \alpha _{ij} & \cdots & 1 \end{array}\right) , \forall i, j = 1,...,s_g. \end{aligned}$$

**Table 1:** Values of $\beta$ for the data generation process in scenario 1 and scenario 2

	$\varvec{\beta }_{\varvec{0}}$	$\varvec{\beta }_{\varvec{1}}$	$\varvec{\beta }_{\varvec{2}}$	$\varvec{\beta }_{\varvec{3}}$	$\varvec{p}^{\varvec{\prime }}$	*n*
Scenario 1	0.00	3.00	−2.00	0.50	22	2000
Scenario 2						
Unbalanced/strong	−2.65	3.60	−2.20	−1.00	197	100
Balanced/strong	0.00	3.60	−4.00	−1.00	197	100
Unbalanced/weak	−2.75	0.60	−2.50	−1.00	197	100
Balanced/weak	0.50	0.60	−2.50	−1.00	197	100

Values of $\beta$ associated with non-informative features are not reported since they were all null. Last two columns are $p'$ number of non-informative features and *n* number of observations

With $s_g$ the size of each group, and $\alpha _{ij}$ the correlation between feature *i* and feature *j*. We considered constant and non-constant levels of correlation within a group. For a constant level of correlation, $\varvec{\Sigma }_g$ is a matrix with 1 on its diagonal and $\alpha _{\max }$ as off-diagonal elements:

$$\begin{aligned} \alpha _{ij} = \left\{ \begin{array}{ll} \alpha _{\max }, \forall i \ne j \\ 1 \textrm{, otherwise}. \end{array} \right. \end{aligned}$$

For a non-constant level of correlation, the level of correlation within a group will depend on the index of features. The correlation between feature *i* and feature *j* within a group will be affected by the value of $|i - j|$:

2 $$\begin{aligned} \alpha _{ij} = \left\{ \begin{array}{ll} \alpha _{\max } \exp \{-w (|i - j| - 1)\}, \forall i \ne j\\ 1 \textrm{, otherwise}. \end{array} \right. \end{aligned}$$

The correlation level $\alpha _{ij}$ will decrease in (2), if the value $|i-j|$ increase. For instance, in a group, two consecutive features will have the maximum correlation level ($|i - j| - 1 = 0$ and $\alpha _{ij} = \alpha _{\max }$). The first and the last feature of a group will have the minimum correlation level ($|i - j| - 1 = s_g - 2$ and $\alpha _{ij} = \alpha _{\max }\exp \{-w(s_g - 2)\}$). Values of the parameter *w* allowed us to control this minimum level of correlation *c* between two features within a group:

$$\begin{aligned} \alpha _{\max } \exp \{-w(|i-j| - 1)\}> c, \end{aligned}$$

if we wish to achieve at least a correlation of *c* between the first and last feature of a group, we set *w* as

$$\begin{aligned} w < \frac{\log (\alpha _{\max }/c)}{s_g - 2}. \end{aligned}$$

The group of features independent from all other features had the identity matrix as a variance-covariance matrix. $\varvec{\Sigma }$ had the following format:

3 $$\begin{aligned} \varvec{\Sigma } = \left( \begin{array}{cccccc} \varvec{\Sigma }_g & \varvec{0} & \cdots & \varvec{0} & \varvec{0} & \varvec{0} \\ \varvec{0} & \varvec{\Sigma }_g & \cdots & \varvec{0} & \varvec{0} & \varvec{0} \\ \vdots & \vdots & \ddots & \vdots & \vdots & \vdots \\ \varvec{0} & \varvec{0} & \cdots & \varvec{\Sigma }_g & \varvec{0} & \varvec{0} \\ \varvec{0} & \varvec{0} & \cdots & \varvec{0} & \varvec{\Sigma }_g & \varvec{0} \\ \varvec{0} & \varvec{0} & \cdots & \varvec{0} & \varvec{0} & I_{s_g \times s_g} \end{array}\right) . \end{aligned}$$

In scenario 1, we considered $n = 2\,000$ observations, and $p' = 22$ non-informative features. In scenario 2, we considered $n = 100$ observations, and $p' = 197$ non-informative features. We also considered different fixed effects $\varvec{\beta }$ for the informative features in order to generate classes with a strong or weak separation. Furthermore, we generated balanced and unbalanced classes. In scenario 1 and scenario 2, $x_1$, $x_2$, and $x_3$ are the only informative features. Table 1 summarises the previous information for scenario 1 and scenario 2, with the values of $\beta$ used in the generation process.

In scenario 3, we considered $n = 2\,000$ observations and the same value of $\beta$ fixed at 1.5, for all informative features. The intercept $\beta _0$ was set to 0. The datasets were generated with 10 groups of 10 features. In the first 5 groups, the first feature was informative and the others were non-informative. Groups 6, 7, 8, and 9 had only non-informative features and group 10 was the independent group with the first feature being informative and the others non-informative. Hence, in scenario 3, $p' = 94$ and $p = 6$. We considered $\varvec{\Sigma }_g$ with constant and non-constant level of dependence with $\alpha _{\max } \in \{ 0.9, 0.6, 0.3\}$ to test high, medium, and low correlation within a group of features. For non-constant level of dependence we set $c \in \{0.35, 0.25, 0.1\}$. Visualisation of $\varvec{\Sigma }$, $\varvec{\Sigma }_g$ and the $\varvec{\beta }$ vector for this scenario are provided in Appendix E.

### Performance indicators

We evaluated feature selection methods primarily based on their predictive power using classification models (AUC, specificity and sensitivity at maximum Youden’s index), the quantity and importance of the selected features (number of informative and non-informative features selected), and the stability of the selection (Jaccard index). Additionally, we measured the execution time for the selection process (runtime).

1. **AUC**: Is a well-known indicator of a classification model’s predictive power. It is computed from the ROC curve, which plots sensitivity against 1-specificity at various probability cutoffs. The AUC is simply the area under this ROC curve and can be interpreted as the probability that the model will rank a randomly chosen positive example higher than a negative one. A higher AUC indicates better model performance.
2. **Sensitivity and specificity**: Using the ROC curve, we also determined the optimal sensitivity and specificity based on Youden’s index. This index is calculated as the point on the ROC curve that maximizes the value of $specificity + sensitivity - 1$.
3. **Feature selection**: In each scenario of the simulation study, we have prior knowledge of which features are truly informative and which are non-informative. For each simulation repetition, we assessed how many of the selected features were informative or non-informative. At the conclusion of the simulation, we calculated the average number of selected informative and non-informative features for each method.
4. **Stability**: The stability of feature selection was evaluated using the average over all pairwise similarity comparisons between the different set of selected features [30]. For a feature selection method we compute its stability over all the repetition using the formula:
  $$\begin{aligned} \text {Stability}(S) = \frac{2}{R(R-1)} \sum\limits_{i = 1}^{R-1}\sum\limits_{j = i+1}^{R}J(S_i, S_j), \end{aligned}$$
  where *S* is the set of all selected features at each iteration by a given method, and *R* is the number of repetitions. The Jaccard index [28], $J(S_i, S_j) = \frac{|S_i \cap S_j|}{|S_i \cup S_j|}$, measures the similarity between the sets of selected features at repetition *i* and *j*. A Jaccard index of 1 means the two sets are identical, while an index of 0 indicates completely different sets.
5. **Runtime**: It is the execution time of the feature selection process in seconds. The simulation study was performed on a device with 16.0Go of RAM, AMD Ryzen 7 5700X 8-Core Processor 3.40 GHz.

### Feature selection methods ranking

Unless a single method outperforms all others across every indicators, it can be challenging to determine which method performs better overall, as each indicator measures a different aspect of performance. To address this, we applied a multiple criteria decision-making (MCDM) method to rank the methods based on their performance across all criteria (in our case, a criteria is a performance indicator). We applied the Technique for Order of Preference by Similarity to Ideal Solution (TOPSIS) method [31, 32]. TOPSIS ranks feature selection methods based on their geometrical distance to an ideal solution, where higher-ranked methods are closer to this ideal and farthest from the negative ideal (the worst solution). Before calculating these distances, the indicators are normalized, and weights are applied based on the relative importance of each indicator as defined by the user. A step-by-step explanation of the TOPSIS score is detailed in Appendix C. In our study, we prioritize the performance of the classification models, specifically their predictive power. This means that the indicators AUC, sensitivity and specificity are given the highest weight. Following that, we assign importance to the number of selected features - preferring methods that select fewer features, provided they achieve similar predictive performance. Finally, the stability of the feature selection methods and their running time are considered of lower importance, and thus, they receive lower weights in the ranking process. Table 2 summarizes the ranking and corresponding weights for each indicator.

**Table 2:** Ranks, weights ($w_i$), and categories of the performance indicators used for evaluating feature selection methods

	AUC	Sensitivity	Specificity	NSIF	NSNIF	Stability	Runtime (s)
Rank	1	2	3	4	5	6	7
Weight $w_i$	0.2500	0.2143	0.1786	0.1429	0.1071	0.0714	0.0357
Category	Benefit	Benefit	Benefit	Benefit	Cost	Benefit	Cost

The Category column specifies whether the indicator is a benefit (higher values preferred) or a cost (lower values preferred)

*Abbreviations: NSIF* Number of Selected Informative Features, *NSNIF* Number of Selected Non-Informative Features

### Results

#### Scenario 1

**Fig. 1:**
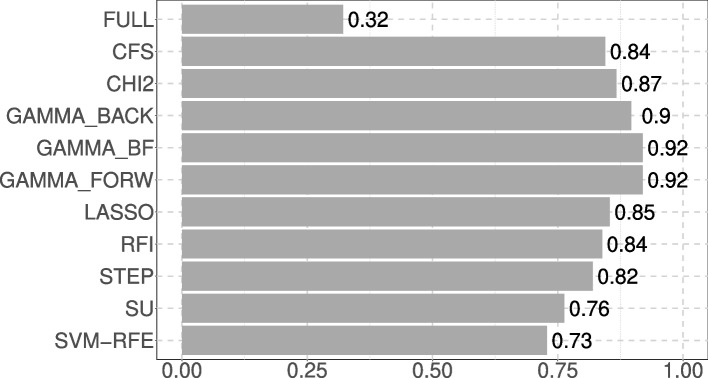
TOPSIS score of the feature selection methods on scenario 1

The results for feature selection methods are presented in Table 3 and TOPSIS scores are presented in Fig. 1. The GAMMA_BF and GAMMA_FORW methods performed similarly, ranking as the top two methods in this scenario. Both demonstrated strong predictive performance, with AUC values of 85.92%, sensitivity of 85.98% and specificity of 86.59%. These methods also selected nearly all of the informative features (an average of 2.92 out of 3), while keeping the number of non-informative features low (1.62 out of 22 on average). The distinction between these methods lies in their execution time that was slightly longer for GAMMA_BF (0.45s against 0.44s for GAMMA_FORW).

**Table 3:** Results for scenario 1, displaying all performance indicators averaged over the 50 repetitions

FSM	AUC	Se	Spe	NSIF	NSNIF	Stability	Runtime (s)	Rank
FULL	85.77	86.11	86.18	3.00	22.00	1.00	0.04	11
CFS	83.76	84.30	84.02	1.82	**0.00**	0.849	0.23	6
CHI2	85.09	86.09	84.88	1.98	**0.00**	**0.980**	**0.16**	4
GAMMA_BACK	**85.92**	85.98	**86.59**	**2.92**	**1.62**	0.805	5.68	3
**GAMMA_BF**	**85.92**	85.98	**86.59**	**2.92**	**1.62**	0.805	0.45	**2**
**GAMMA_FORW**	**85.92**	85.98	**86.59**	**2.92**	**1.62**	0.805	0.44	**1**
LASSO	84.02	84.48	84.21	2.90	2.26	0.504	2.38	5
RFI	85.25	**86.21**	85.05	2.00	**0.00**	**1.00**	9.22	7
STEP	85.87	**86.41**	86.05	**3.00**	3.16	0.404	1.75	8
SU	77.28	78.18	77.27	1.08	**0.00**	0.925	**0.14**	9
SVM-RFE	85.91	86.16	**86.42**	**3.00**	2.44	0.489	23.36	10

The best value of each indicator is highlighted with bold and underline text. The second-best value is highlighted in bold. The indicators for the FULL model are not highlighted in the table since the focus of the study is to compare the feature selection methods

*Abbreviations: FSM* Feature Selection Methods, *Se* Sensitivity, *Spe* Specificity, *NSIF* Number of Selected Informative Features, *NSNIF* Number of Selected Non-Informative Features

Other methods also showed good performance. CFS, CHI2, RFI, and SU were able to select only informative features, but fewer than the $\gamma$-metric based methods, resulting in lower predictive power and rankings (6th, 4th, 7th, and 9th for CFS, CHI2, RFI, and SU, respectively). Notably, RFI exhibited excellent stability, with a Jaccard index of 1.00, indicating that it consistently selected the same two informative features. However, RFI was unable to select all the informative features, limiting its predictive performance.

STEP and SVM-RFE were able to select all informative features in each iteration but also included more non-informative features. SU performed the worst, with an average of only 1.08 informative features selected and significantly lower predictive power (AUC = 77.28%, sensitivity = 78.18%, and specificity = 77.27%).

Figure 1 illustrates how closely the methods actually performed in this scenario.

#### Scenario 2

**Fig. 2:**
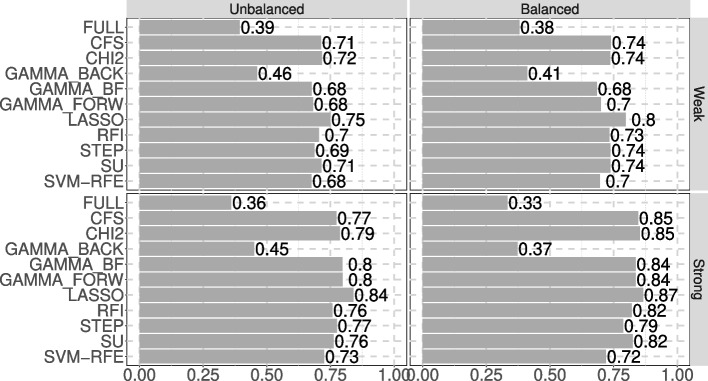
TOPSIS scores of the feature selection methods on scenario 2

Results for all feature selection methods and indicators (for 200 features) are presented in Table 4, with TOPSIS scores shown in Fig. 2. In this scenario, LASSO consistently emerged as the best feature selection method across all cases (as reflected in its TOPSIS score in Fig. 2). In the strong separation case, GAMMA_BF and GAMMA_FORW also performed well, ranking 2nd and 3rd with unbalanced classes, and 4th and 5th with balanced classes, respectively. Both methods achieved the second-best AUC values (81.46% for unbalanced and 87.46% for balanced classes) and the highest sensitivity (88.70% for unbalanced classes).

**Table 4:** Results for scenario 2, displaying all performance indicators averaged over 50 repetitions

	Unbalanced classes									Balanced classes							
FSM	AUC	Se	Spe	NSIF	NSNIF	Stability	Runtime	Rank	FSM	AUC	Se	Spe	NSIF	NSINF	Stability	Runtime	Rank
Weak separation																	
FULL	53.23	62.19	49.75	3.00	197.00	1.00	0.01	11	FULL	53.53	57.75	52.52	3.00	197.00	1.00	0.01	11
CFS	71.96	**88.23**	78.80	1.18	0.36	0.638	1.39	4	**CFS**	77.59	80.80	81.52	1.24	0.28	0.681	1.77	**2**
**CHI2**	**72.06**	**88.38**	78.94	1.22	0.38	0.616	**0.36**	**2**	CHI2	**77.61**	**81.31**	81.11	1.32	0.44	0.552	**0.48**	5
GAMMA_BACK	54.71	61.86	56.23	**2.60**	93.62	0.317	271.90	10	GAMMA_BACK	53.16	58.03	54.18	**2.82**	115.22	0.422	299.86	10
GAMMA_BF	68.27	78.59	74.40	1.82	17.52	0.119	60.52	8	GAMMA_BF	70.33	73.12	73.91	**2.50**	28.82	0.130	136.31	9
GAMMA_FORW	68.27	78.59	74.40	1.82	17.52	0.119	26.41	7	GAMMA_FORW	70.33	73.12	73.91	**2.50**	28.82	0.130	60.18	7
**LASSO**	**72.15**	87.08	**79.84**	1.54	0.92	0.619	1.85	**1**	**LASSO**	**78.34**	**81.17**	**81.81**	2.00	1.08	0.510	2.41	**1**
RFI	70.98	86.33	77.21	1.02	**0.14**	**0.805**	**0.45**	5	RFI	77.23	80.27	80.86	1.04	**0.00**	**0.961**	0.68	6
STEP	70.82	58.12	**86.06**	1.94	5.10	0.136	3.60	6	STEP	73.72	76.71	72.72	2.34	7.36	0.137	5.70	3
SU	71.90	88.01	78.95	1.18	**0.34**	**0.659**	**0.45**	3	SU	77.46	80.21	**81.61**	1.18	**0.26**	**0.733**	**0.61**	4
SVM-RFE	64.34	65.03	75.19	**2.12**	19.32	0.090	10.07	9	SVM-RFE	67.30	71.72	69.38	2.48	24.86	0.104	14.63	8
Strong separation																	
FULL	54.36	60.03	53.04	3.00	197.00	1.00	0.01	11	FULL	54.44	61.21	53.30	3.00	197.00	1.00	0.01	11
CFS	78.82	86.43	84.10	1.54	0.36	0.586	1.77	6	CFS	**87.86**	**90.52**	90.37	2.04	**0.40**	0.711	2.49	3
CHI2	79.81	87.23	85.17	1.70	0.44	0.561	**0.38**	4	**CHI2**	**88.41**	**91.12**	90.74	2.10	0.46	**0.713**	**0.43**	**2**
GAMMA_BACK	56.49	61.30	59.48	**2.88**	92.36	0.316	274.23	10	GAMMA_BACK	55.45	61.06	56.06	**2.80**	107.92	0.382	268.72	10
GAMMA_BF	**81.46**	**88.70**	84.96	2.30	4.50	0.357	17.85	3	GAMMA_BF	**87.86**	89.33	**91.19**	2.24	1.04	0.525	7.36	5
**GAMMA_FORW**	**81.46**	**88.70**	84.96	2.30	4.50	0.357	10.09	**2**	GAMMA_FORW	**87.86**	89.33	**91.19**	2.24	1.04	0.525	6.05	4
**LASSO**	**82.84**	**87.94**	**87.41**	2.36	2.02	0.536	1.95	**1**	**LASSO**	87.74	89.04	**91.27**	2.26	1.30	0.698	2.17	**1**
RFI	76.13	85.21	81.39	1.22	**0.00**	**0.819**	0.51	8	RFI	84.94	88.19	86.98	1.78	**0.00**	**0.780**	0.61	7
STEP	81.07	76.48	**88.12**	2.48	3.58	0.243	3.53	5	STEP	83.76	84.88	84.33	**2.56**	3.90	0.235	4.12	8
SU	77.44	85.75	82.50	1.36	**0.22**	**0.668**	**0.48**	7	SU	86.58	89.97	88.29	1.92	0.42	0.65	**0.53**	6
SVM-RFE	72.16	76.14	78.41	**2.68**	16.86	0.116	10.48	9	SVM-RFE	75.52	76.71	79.98	2.52	18.26	0.110	11.54	9

The best value of each indicator is highlighted with bold and underline. The second-best value is highlighted in bold. The indicators for the FULL model are not highlighted in the table since the focus of the study is to compare the feature selection methods

*Abbreviations: FSM* Feature Selection Methods, *Se* Sensitivity, *Spe* Specificity, *NSIF* Number of Selected Informative Features, *NSNIF* Number of Selected Non-Informative Features

For balanced classes, CFS and CHI2 were ranked 3rd and 2nd, respectively. These methods excelled at ignoring non-informative features (averaging 0.40 for CFS and 0.46 for CHI2), achieving the best and second-best AUC values (87.86% for CFS and 88.41% for CHI2) and sensitivity (90.52% for CFS and 91.12% for CHI2).

In the case of weak separation between classes, all methods demonstrated lower predictive performance, with the highest AUC at 72.15% (LASSO). For unbalanced classes, GAMMA_BF and GAMMA_FORW selected more non-informative features (17.52 on average out of 197) and fewer informative ones (1.82 on average out of 3). GAMMA_BACK managed to select the most informative features (2.60 on average) but also included a high number of non-informative features (93.62 on average). SVM-RFE exhibited a similar pattern, selecting many non-informative features (19.32 on average for unbalanced classes, 24.86 for balanced classes) and a moderate number of informative features (2.12 for unbalanced classes and 2.48 for balanced classes).

As shown in Fig. 2, LASSO remains the top-performing method, especially in cases with strong classes separation, with GAMMA_BF and GAMMA_FORW consistently close behind in strong separation scenarios.

#### Scenario 3

**Fig. 3:**
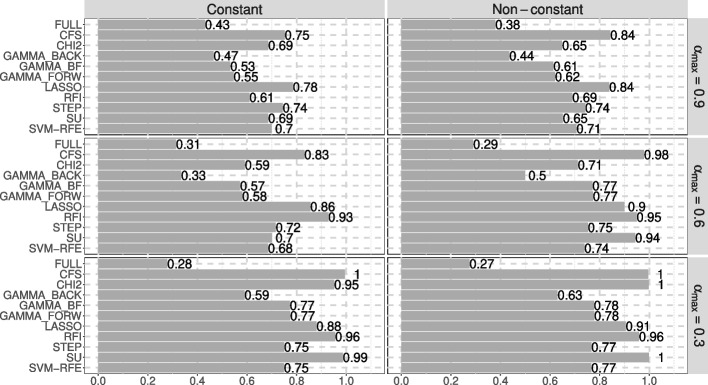
TOPSIS scores of the feature selection methods on scenario 3

Results for all feature selection methods and indicators are presented in Table 5, with TOPSIS scores shown in Fig. 3.

**Table 5:** Results for scenario 3, displaying all indicators average over the 50 repetitions

	Constant correlation									Non constant correlation							
FSM	AUC	Se	Spe	NSIF	NSNIF	Stability	Runtime	Rank	FSM	AUC	Se	Spe	NSIF	NSNIF	Stability	Runtime	Rank
$$\alpha _{\max } = 0.9$$																	
FULL	84.66	84.80	85.18	6.00	94.00	1.00	0.03	11	FULL	85.64	84.46	84.57	6.00	94.00	1.00	0.00	11
**CFS**	**85.68**	**86.56**	85.54	**5.94**	19.82	0.393	9.46	**2**	**CFS**	**85.81**	**86.09**	86.29	**6.00**	8.08	**0.625**	4.20	**1**
CHI2	85.40	85.95	85.47	**6.00**	45.00	**1.00**	**0.58**	5	CHI2	85.40	85.36	**86.34**	**6.00**	41.42	**0.897**	**0.52**	7
GAMMA_BACK	84.90	85.26	85.16	**6.00**	64.24	0.712	68.64	10	GAMMA_BACK	84.93	85.41	85.21	**6.00**	57.16	0.570	73.57	10
GAMMA_BF	84.95	85.45	85.23	**6.00**	57.56	**0.725**	42.92	9	GAMMA_BF	85.29	85.69	85.64	**6.00**	35.48	0.408	25.94	9
GAMMA_FORW	84.95	85.45	85.23	**6.00**	57.56	**0.725**	31.95	8	GAMMA_FORW	85.29	85.69	85.64	**6.00**	35.48	0.408	18.89	8
**LASSO**	**85.62**	86.04	**85.79**	5.92	**7.24**	0.373	2.99	**1**	**LASSO**	85.48	**85.90**	85.89	**5.94**	**3.48**	0.562	2.91	**2**
RFI	69.82	69.90	70.74	2.70	15.30	0.551	18.36	7	RFI	71.63	74.33	69.95	3.00	**0.32**	0.489	17.72	5
STEP	85.46	**86.40**	85.16	**6.00**	11.08	0.283	18.71	3	STEP	85.33	84.94	**86.57**	**6.00**	9.54	0.299	15.39	3
SU	85.40	85.95	85.47	**6.00**	45.00	**1.00**	**0.68**	6	SU	80.92	80.24	82.47	5.02	33.10	0.583	**0.62**	6
SVM-RFE	85.50	85.75	**86.07**	**6.00**	**10.02**	0.310	55.89	4	SVM-RFE	**85.55**	85.73	86.24	**6.00**	8.28	0.348	55.58	4
$$\alpha _{\max } = 0.6$$																	
FULL	84.60	85.56	84.47	6.00	94.00	1.00	0.00	11	FULL	84.72	84.72	85.50	6.00	94.00	1.00	0.00	11
CFS	**85.94**	85.89	**86.68**	**6.00**	5.96	0.559	3.94	3	**CFS**	**86.01**	**86.06**	**86.61**	**6.00**	**0.24**	**0.935**	1.88	**1**
CHI2	85.45	**86.20**	85.39	**6.00**	44.98	**0.999**	**0.52**	7	CHI2	85.72	85.84	86.19	**6.00**	27.76	0.655	**0.51**	9
GAMMA_BACK	84.83	85.77	84.68	**6.00**	62.52	0.670	68.40	10	GAMMA_BACK	85.24	85.16	85.98	**6.00**	40.44	0.383	94.79	10
GAMMA_BF	85.31	85.40	85.98	**6.00**	36.48	0.412	27.75	9	GAMMA_BF	85.61	85.60	86.37	**6.00**	16.92	0.473	13.93	6
GAMMA_FORW	85.31	85.40	85.98	**6.00**	36.48	0.412	20.63	8	GAMMA_FORW	85.61	85.60	86.37	**6.00**	16.92	0.473	10.29	5
**LASSO**	85.22	85.29	85.84	**5.88**	**2.76**	0.607	2.42	**2**	LASSO	85.45	85.51	86.08	5.92	2.06	0.669	2.42	4
**RFI**	**86.03**	**85.94**	**86.78**	**6.00**	**0.00**	**1.00**	17.55	**1**	**RFI**	**85.91**	**85.99**	**86.53**	**5.98**	**0.00**	**0.993**	17.03	**2**
STEP	85.41	85.42	86.08	**6.00**	12.56	0.267	18.60	4	STEP	85.28	85.60	85.75	**6.00**	13.26	0.261	18.25	7
SU	84.28	83.98	85.25	5.72	25.18	0.511	**0.62**	5	SU	84.20	83.99	85.16	5.64	0.56	0.852	**0.61**	3
SVM-RFE	85.59	85.91	85.96	**6.00**	8.68	0.325	59.76	6	SVM-RFE	85.52	85.54	86.28	**6.00**	9.42	0.315	58.75	8
$$\alpha _{\max } = 0.3$$																	
FULL	84.73	84.38	85.84	6.00	94.00	1.00	0.00	11	FULL	84.64	84.95	85.07	6.00	94.00	1.000	0.00	11
**CFS**	**86.23**	**87.02**	86.07	**6.00**	**0.00**	**1.000**	1.81	**1**	CFS	**86.05**	**86.18**	**86.67**	**6.00**	**0.00**	**1.000**	1.80	3
CHI2	**86.19**	**86.87**	**86.17**	**6.00**	**1.92**	0.841	**0.50**	4	**CHI2**	**86.04**	**86.23**	**86.61**	**6.00**	**0.02**	**0.994**	**0.51**	**2**
GAMMA_BACK	85.44	85.69	85.93	**6.00**	28.70	0.310	109.10	10	GAMMA_BACK	85.45	85.68	85.91	**6.00**	23.14	0.325	118.05	10
GAMMA_BF	85.71	86.01	**86.16**	**6.00**	17.10	0.440	13.97	7	GAMMA_BF	85.56	85.69	86.15	**6.00**	15.88	0.371	12.49	7
GAMMA_FORW	85.71	86.01	**86.16**	**6.00**	17.10	0.440	10.33	6	GAMMA_FORW	85.56	85.69	86.15	**6.00**	15.88	0.371	9.10	6
LASSO	85.85	86.63	85.71	**5.98**	4.56	0.587	2.38	5	LASSO	85.36	85.84	85.52	**5.90**	2.40	0.646	2.40	5
RFI	**86.23**	**87.02**	86.07	**6.00**	**0.00**	**1.000**	17.43	3	RFI	**86.05**	**86.18**	**86.67**	**6.00**	**0.00**	**1.000**	17.50	4
STEP	85.28	85.96	85.33	**6.00**	15.56	0.251	21.37	8	STEP	85.27	85.51	85.85	**6.00**	14.14	0.255	19.87	8
**SU**	85.73	86.39	85.77	5.90	**0.00**	**0.967**	**0.62**	**2**	**SU**	**86.05**	**86.18**	**86.67**	**6.00**	**0.00**	**1.000**	**0.59**	**1**
SVM-RFE	85.54	85.90	85.93	**6.00**	10.64	0.291	60.70	9	SVM-RFE	85.47	86.01	85.74	**6.00**	9.52	0.305	60.91	9

The best value of each indicator is highlighted with bold and underline. The second-best value is highlighted in bold. The indicators for the FULL model are not highlighted in the table since the focus of the study is to compare the feature selection methods

*Abbreviations: FSM* Feature Selection Methods, *Se* Sensitivity, *Spe* Specificity, *NSIF* Number of Selected Informative Features, *NSNIF* Number of Selected Non-Informative Features

**Constant correlation:** First, let’s consider the case where the correlation is constant (the left side of Table 5). In the case of a strong correlation $\alpha _{max} = 0.9$, LASSO was ranked 1st, followed by CFS in 2nd. Both methods achieved an AUC above 85%, with CFS and LASSO reaching 85.68% and 85.62%, respectively. They also had high sensitivity values: 86.56% for CFS and 86.04% for LASSO, as well as among the best specificity (85.54% for CFS and 85.79% for LASSO). In this scenario, most feature selection methods were able to select all the informative features, but none could fully exclude the non-informative ones. GAMMA_BF and GAMMA_FORW were ranked 9th and 8th, respectively. Both selected all the informative features, but they also selected a significant number of non-informative features (an average of 57.56 out of 94 features). RFI was less efficient at selecting informative features under strong correlation (2.70 on average out of 6) and exhibited the worst predictive performance (AUC = 69.82%, sensitivity = 69.90%, and specificity = 70.74%).

When the correlation level $\alpha _{max}$ was lower ($\alpha _{max} = 0.6$), RFI performed much better compared to the strong correlation case. It was able to select all the informative features (6.00 on average) and excluded all the non-informative ones. RFI also achieved the best performance in terms of AUC (86.03%), specificity (86.78%), and the second-best sensitivity (85.94%). Additionally, RFI demonstrated perfect stability (1.00), consistently selecting only informative features. LASSO and CFS ranked 2nd and 3rd, respectively. LASSO selected only a few non-informative features (2.76 on average), although it also selected slightly fewer informative features. CFS had the second-best AUC (85.94%) and specificity (86.68%). GAMMA_BF and GAMMA_FORW performed well in terms of predictive metrics (AUC = 85.31%, sensitivity = 85.40%, and specificity = 85.98%), but they selected some non-informative features (36.48 on average for both).

At a correlation level $\alpha _{max}$ of 0.3, CFS ranked 1st with an AUC of 86.23% (tied with RFI) and a sensitivity of 87.02%. CFS, SU, and RFI were the only methods that selected only informative features, ranking 1st, 2nd, and 3rd, respectively. In this case, GAMMA_BF and GAMMA_FORW improved rankings and achieved the second-best specificity (86.16%).

**Non-constant correlation:** Next, we look at the case of non-constant correlation (the right side of Table 5). For a strong correlation level ($\alpha _{max} = 0.9$), CFS and LASSO once again ranked 1st and 2nd, respectively, with CFS achieving the best AUC (85.81%) and sensitivity (86.09%). In this setting, the feature selection methods generally selected fewer non-informative features. For instance, GAMMA_BF and GAMMA_FORW selected an average of 35.48 non-informative features compared to 57.56 in the constant correlation case. Both GAMMA_BF and GAMMA_FORW achieved over 85% in AUC, sensitivity, and specificity. RFI selected very few non-informative features (0.32 on average) but was only able to select 3.00 informative features, resulting in the lowest predictive performance (AUC = 71.63%, sensitivity = 74.33%, and specificity = 69.95%).

For a lower correlation level ($\alpha _{max} = 0.6$), CFS clearly ranked 1st, demonstrating the highest predictive performance, with all indicators exceeding 86%. RFI ranked 2nd with strong AUC (85.91%), sensitivity (85.99%), and specificity (86.53%). Moreover, RFI did not select any non-informative features and maintained a high stability score (0.993). GAMMA_BF and GAMMA_FORW ranked 6th and 5th, with a high specificity (86.37% for both) and an AUC of 85.61%. However, both methods selected a similar number of non-informative features (16.92 on average).

At the lowest correlation level ($\alpha _{max} = 0.3$), SU, RFI, and CFS achieved similar resulats, except for differences in running time. They ranked 1st, 4th, and 3rd, respectively, with an AUC of 86.05%, sensitivity of 86.18%, and specificity of 86.67%. These three methods successfully selected all and only the informative features. CHI2 had comparable results and ranked 2nd, mainly thanks to its short execution time (0.51s on average). GAMMA_BF and GAMMA_FORW ranked 7th and 6th, respectively, with an average of 15.88 non-informative features selected, while still selecting all informative features.

## Application

To evaluate the performance of the three proposed novel $\gamma$-metric feature selection methods, we compared each method’s ability to select the most relevant features for distinguishing between AF and NSR using Holter-based ECG data. This evaluation was based on the classification performance of models built with the features selected by each method.

The Holter ECG recordings used in this study were from patients who exhibited only one type of cardiac activity - either AF or NSR. These data, which were previously collected as part of routine medical care, were obtained from the Department of Cardiology and Rhythmology at Marseille University Hospital Center (Timone Hospital) in France. The sample consisted of 34 files, each corresponding to a 24-hour Holter recording for and individual patient. Recordings were conducted between November 2016 and February 2017. The sample included 18 men and 16 women, with a median age of 62 years. Among these patients, 11 (32.4%) had episodes of AF.

### Data

The recordings consisted of RR-intervals, or beat-to-beat time intervals. Each patient’s recording was segmented into a total 41 661 segments, each lasting 60 seconds. For each segment, we calculated various heart rhythm variability indicators, as outlined by [33]. These included: (i) the standard deviation of all RR intervals (SDNN); (ii) the standard deviation of the averages of 5-second RR intervals (SDANN); (iii) the mean of the standard deviations of 5-second RR intervals (SDNNidx); (iv) the percentage of differences between successive RR intervals greater than 50 ms (pNN50); (v) the standard deviation of successive differences (SDSD); (vi) the root-mean-square of successive differences (RMSSD); (vii) the interquartile range of the differences between successive RR intervals (IRRR); (viii) the median of the absolute differences between adjacent RR intervals (MADRR); (ix) the triangular interpolation of the RR interval histogram (TINN); (x) the integral of the RR interval histogram density divided by its height (HRV.index); (xi) the means (denoted mn.0df to mn.2df); and (xii) the standard deviations (denoted sd.0df to sd.2df) of the RR-intervals derivatives (up to the 2nd order). Altogether, the dataset included 16 features, which are described in Table 6 for the training sample.

**Table 6:** Description of the training sample for AF and NSR. Mean and standard (in brackets) deviations are displayed for each feature

	AF (N = $\textbf{6}\,\textbf{087}$)	NSR (N = $\textbf{16}\,\textbf{712}$)	*p*-value $\varvec{\dagger }$
mn.0df (mean (SD))	0.73 (0.17)	0.89 (0.14)	$<0.001$
sd.0df (mean (SD))	0.14 (0.04)	0.05 (0.03)	$<0.001$
mn.1df (mean (SD))	−0.04 (0.02)	0.00 (0.01)	$<0.001$
sd.1df (mean (SD))	0.29 (0.08)	0.04 (0.06)	$<0.001$
mn.2df (mean (SD))	0.13 (0.08)	0.01 (0.03)	$<0.001$
sd.2df (mean (SD))	0.79 (0.35)	0.10 (0.24)	$<0.001$
SDNN (mean (SD))	144.06 (35.58)	49.37 (34.62)	$<0.001$
SDANN (mean (SD))	57.84 (25.01)	38.16 (28.03)	$<0.001$
SDNNIDX (mean (SD))	136.12 (32.62)	27.01 (20.52)	$<0.001$
pNN50 (mean (SD))	76.79 (7.23)	11.68 (16.09)	$<0.001$
SDSD (mean (SD))	201.73 (49.18)	36.81 (38.55)	$<0.001$
RMSSD (mean (SD))	200.48 (48.76)	36.54 (38.27)	$<0.001$
IRRR (mean (SD))	186.24 (48.08)	60.91 (42.72)	$<0.001$
MADRR (mean (SD))	129.02 (33.84)	19.58 (15.68)	$<0.001$
TINN (mean (SD))	256.39 (54.54)	121.61 (46.07)	$<0.001$
HRV.index (mean (SD))	16.41 (3.49)	7.78 (2.95)	$<0.001$

$^{\dagger }$ The *p*-value is based on the Mann-Whitney U test

### Feature selection and classification

In this healthcare-focused application, the dataset was divided into a training sample and a validation sample, using 60% of the dataset for the training sample. Sampling was done so segments from the same patient was exclusively in the training sample or exclusively in the validation sample. Feature selection was performed on the training sample for each feature selection method, and the selected features were then used to build a logistic regression classifier. The performance of each logistic regression classifier was subsequently evaluated on both the training and validation samples.

Since the truly informative features were not known beforehand, it was not feasible to define if the selected features were truly informative or not. Hence the evaluation of the performances rely solely on the AUC, the sensitivity and specificity (at maximum Youden index), the number of selected features in total, and the running time of the feature selection process. Feature selection was run only once, so no stability index could be computed. For all the above reasons, TOPSIS score was not computed on the healthcare-case application. For this study, AF was treated as the positive case. Sensitivity, in this context, represents the percentage of correctly predicted AF cases, while specificity represents the percentage of correctly predicted NSR cases.

### Results

The results for discriminating AF from NSR are presented in Tables 7 and 8 for the training and validation samples, respectively. The results from the FULL method highlight the importance of including a feature selection step. On the training sample, the model built with all features showed near-perfect sensitivity and specificity (99.61% and 99.83%, respectively), but its performance dropped significantly when applied to new data. On the validation sample, the FULL model’s sensitivity decreased to 95.08%, while its specificity remained high at 99.7%.

**Table 7:** Training sample results for AF detection

FSM	AUC	Sensitivity	Specificity	NSF	Runtime (s)
FULL	99.72	99.61	99.83	16	0.00
CFS	99.95	99.46	99.41	3	0.11
CHI2	99.87	99.77	99.96	15	0.83
GAMMA_BACK	99.99	99.95	99.90	7	2.12
GAMMA_BF	99.99	99.95	99.90	7	0.91
GAMMA_FORW	99.99	99.95	99.90	7	0.80
LASSO	99.73	99.51	99.95	7	20.84
RFI	78.42	71.35	86.93	1	178.29
STEP	99.99	99.98	99.95	6	52.84
SU	99.87	99.77	99.96	15	0.87
SVM-RFE	99.81	99.66	99.96	14	43.97

*NSF* Number of Selected Features

**Table 8:** Validation sample results for AF detection

FSM	AUC	Sensitivity	Specificity	NSF	Runtime (s)
FULL	95.48	91.26	99.7	16	0.00
CFS	**99.51**	**98.61**	96.33	**3**	**0.11**
CHI2	95.28	90.90	**99.67**	15	0.83
GAMMA_BACK	**98.74**	**95.08**	97.40	7	2.12
GAMMA_BF	**98.74**	**95.08**	97.40	7	0.91
GAMMA_FORW	**98.74**	**95.08**	97.40	7	**0.80**
LASSO	91.27	82.87	99.66	7	20.84
RFI	66.22	56.52	79.36	**1**	178.29
STEP	98.27	94.75	98.07	6	52.84
SU	95.28	90.90	99.67	15	0.87
SVM-RFE	93.46	87.17	**99.75**	14	43.97

The best value of each indicator is highlighted with bold and underline. The second-best value is highlighted in bold. The indicators for the FULL model are not highlighted in the table since the focus of the study is to compare the feature selection methods

*Abbreviation: NSF* Number of Selected Features

 The CHI2, SU, and SVM-RFE methods selected nearly all features and achieved very high specificity (99.67%, 99.67%, and 99.75%, respectively), but their sensitivity was considerably lower (90.90%, 90.90%, and 87.17%). In contrast, the RFI method, which selected only one feature, performed the worst on the validation sample, with an AUC of 66.22%, sensitivity of 56.52%, and specificity of 79.36%. The $\gamma$-metric based methods - GAMMA_BACK, GAMMA_BF, and GAMMA_FORW - had similar results. They achieved the second-highest AUC (98.74%) and sensitivity (95.08%) and maintained a high specificity of 97.4%. LASSO selected seven features but showed lower sensitivity (82.87%) and AUC (91.27%). Finally, the CFS method delivered the best overall results, selecting three features and achieving an AUC of 99.51%, sensitivity of 98.61%, and specificity of 96.33%.

## Discussions

In this article, we proposed a novel multivariate filter methodology for feature selection, based on three distinct methods that utilize the $\gamma$-metric as an evaluation function for classification. These methods - GAMMA_BACK, GAMMA_BF, and GAMMA_FORW - differ in their search direction. Unlike the univariate methods presented by [9], the methods explored here are multivariate. We also incorporated shrinkage estimation of the covariance matrix into the $\gamma$-metric calculation and compared these methods to seven traditional feature selection methods: CFS, CHI2, LASSO, RFI, STEP, SU, and SVM-RFE. Both simulation studies and real-world data on AF detection were used for comparison. The three $\gamma$-metric based methods effectively identified features with non-null effects, although they were less efficient at excluding non-informative features. GAMMA_BACK tended to select more features compared to GAMMA_BF and GAMMA_FORW. Additionally, the $\gamma$-metric could only be computed for numerical features, limiting the feature selection to numerical data.

The capacity of the $\gamma$-metric based methods to detect informative features was illustrated in all scenario and also in the application, Table 9 summarizes the best results and key conclusions from each scenario. When the informative features had varying effect size (Scenario 1), the $\gamma$-metric based methods consistently selected the three informative features, including $x_3$, which had the smallest $\beta$ coefficient. Only STEP and SVM-RFE selected more informative features in this scenario. GAMMA_BACK, GAMMA_BF, and GAMMA_FORW outperformed the other methods and were ranked 1st, 2nd and 3rd according to the TOPSIS score. We tested the capacity of the methods to perform feature selection in the cases where the number of features was greater than the number of observations (Scenario 2), with both balanced and unbalanced classes, as well as weak and strong class separation. In strong separation cases, GAMMA_BF and GAMMA_FORW successfully selected most of the informative features and only a few non-informative ones, even achieving the 3rd and 2nd rank respectively for unbalanced data and 5th and 4th rank with balanced classes. We tested if the methods were able to disregard non-informative features that were correlated to informative features (Scenario 3) with different levels of correlation. The $\gamma$-metric methods consistently selected all informative features. GAMMA_BF and GAMMA_FORW were more successful at excluding non-informative features when the correlation level was low. In the healthcare application, GAMMA_BACK, GAMMA_BF and GAMMA_FORW demonstrated strong predictive performance, selecting seven features, striking a balance between methods that selected almost all features but had lower predictive performance (e.g., SVM-RFE with 87.17% sensitivity vs. 95.08% for $\gamma$-metric based methods) and RFI, which selected only one feature but had a very low sensitivity of 56.52%, compared to 95.08% for the proposed methods. Only CFS outperformed them, selecting three features and achieving a high AUC of 99.51%, sensitivity of 98.61%, and specificity of 96.33%. For AF detection, [34] reported the accuracy of general practitioners (92% specificity and 80% sensitivity) and practice nurses (85% specificity and 77% sensitivity) in distinguishing AF from NSR cases (99% specificity and 83% sensitivity). Similarly, [35] described the performance of a computer-based algorithm for diagnosing primary cardiac rhythms, including AF, with a specificity of 98.9% and sensitivity of 90.8% for AF diagnosis.

**Table 9:** Summary of the best results and key conclusions for each scenario in the simulation study

	Best results	Conclusions
**Scenario 1:** Informative features with different effect size.	$\gamma$-metric methods selected almost every time each informative features and very few non-informative features.	In classical feature selection tasks, the proposed methods outperformed the other methods.
**Scenario 2:** Large dataset with balanced/unbalanced classes and weak/strong separation.	GAMMA_FORW and GAMMA_BF could select very few features among which mostly the informative ones for the strong separation cases.	GAMMA_FORW and GAMMA_BF were able to perform well with few observations w.r.t the number of features.
**Scenario 3:** Different levels of correlation between informative and non-informative features.	GAMMA_FORW and GAMMA_BF could select all informative features and less non-informative features with low correlation levels.	GAMMA_BF and GAMMA_FORW were able to disregard non-informative features that were less correlated to the informative features.

Despite their effectiveness, the three methods based on the proposed $\gamma$-metric methods can still be improved, as they have certain limitations. In all scenarios, they selected some non-informative features, with GAMMA_BACK being particularly prone to this. In Scenario 1, the number of selected non-informative features was very low and did not impact the predictive performance of the models. This was more pronounced in Scenario 2, where GAMMA_BACK selected nearly half the features, compared to GAMMA_BF and GAMMA_FORW, which performed better in cases of strong class separation. This is likely because the $\gamma$-metric value is predominantly influenced by informative features, and adding or removing non-informative features does not significantly affect its value. GAMMA_BACK, a backward search method, may terminate prematurely, retaining non-informative features if removing them doesn’t drastically change the $\gamma$-metric. In contrast, GAMMA_FORW’s forward search direction stops adding features once all informative ones are selected, which helps avoid non-informative features. In both methods, the $\gamma$-metric is primarily impacted by the inclusion or exclusion of informatives features. Another limitation is the restriction to numerical features, as the $\gamma$-metric requires covariance matrix calculations.

Future work could address these limitations. One potential improvement is to introduce a penalty in the $\gamma$-metric calculation, based on the number of features in the set. This would favor smaller feature sets and help the algorithm ignore non-informative features more effectively. The $\gamma$-metric would then account for both a distance criterion and the dimensionality of the feature subsets. Additionally, exploring alternative search directions may improve performance. As demonstrated, search strategies have a significant impact on results, even when using the same evaluation function. Future search could explore method like genetic algorithms [36] combined with the $\gamma$-metric to avoid local optima by introducing randomness during the search.

Lastly, expanding the $\gamma$-metric to handle qualitative features would be a valuable direction for future research. For example, existing methods for calculating covariance for categorical features could be adapted [37] proposed a variance definition for categorical features, while [38] discussed the use of polychoric correlation [39] for ordinal features in principal component analysis.

## Conclusions

Based on the results from both the simulation study and the healthcare application, the proposed feature selection methods utilizing the $\gamma$-metric as an evaluation function were effective in identifying informative features. Combining the $\gamma$-metric with a forward search strategy, such as in GAMMA_FORW, produced excellent results in traditional feature selection tasks and reasonable performance in scenarios with high feature correlation and large datasets. However, the backward search method, GAMMA_BACK, was more prone to getting stuck in local optima, resulting in the selection of more features than necessary.

## Figures and Tables

**Fig. 4:**
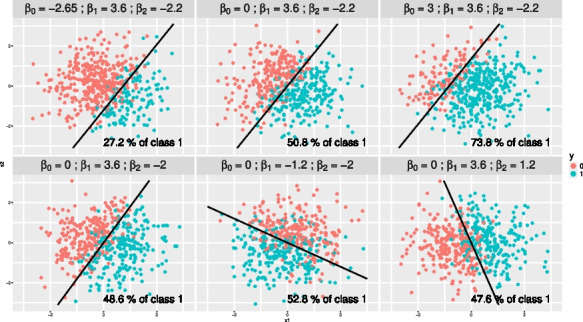
Illustration of data generation with different values of $\varvec{\beta }$ and two features. The straight black line shows the coordinates of points where $\rho _i = 1/2$. The percentage of observations labeled as 1 at the end of the generation procedure is displayed in the bottom-right corner. The top panels depict the class of each observation for varying values of $\beta _0$, while $\beta _1$ and $\beta _2$ are held constant. The bottom panels show the class of each observation with $\beta _0$ fixed at zero and, varying values of $\beta _1$ and $\beta _2$

**Fig. 5:**
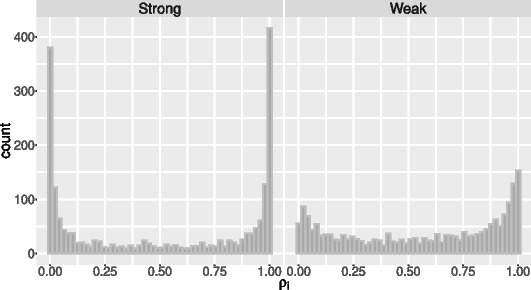
Distribution of the probability $\rho _i$ based on the chosen $\varvec{\beta }$ values, illustrating strong (left panel) and weak (right panel) separation between classes

**Fig. 6:**
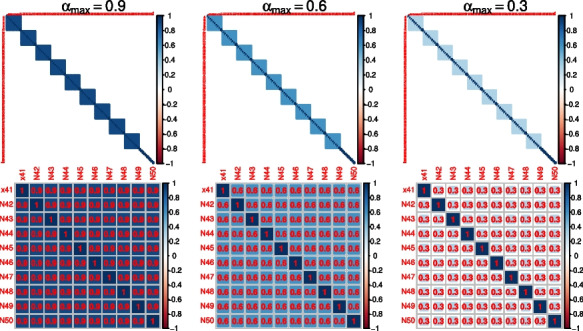
The $\varvec{\Sigma }$ matrix (top panels) used for data generation in scenario 3, illustrating constant correlation at different levels. The $\varvec{\Sigma }_g$ matrix (bottom panels) represents the correlation structure within a group of features

**Fig. 7:**
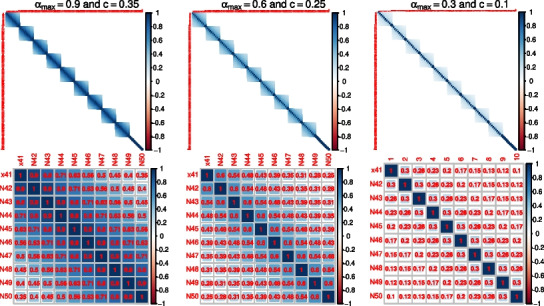
The $\varvec{\Sigma }$ matrix (top panels) used for data generation in scenario 3, showing non-constant correlations at different levels. The $\varvec{\Sigma }_g$ matrix (bottom panels) represents the correlation structure within a group of features

**Fig. 8:**
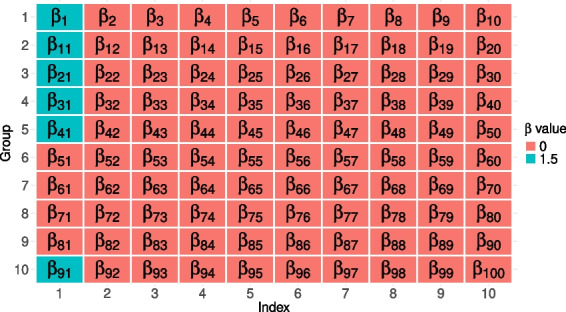
Illustration of the $\varvec{\beta }$ vector used in the generation process for scenario 3. Blue cells indicate $\beta$ coefficients corresponding to informative features, while red cells indicate coefficients corresponding to non-informative features

## Data Availability

The dataset analyzed for the healthcare application is available from the corresponding author upon reasonable request. The R code used to generate the simulation study datasets, as well as to perform the analyses, and produce the tables and figures in this manuscript, is available in the repository https://github.com/NicolasNgo/Multivariate-filter-methods-for-feature-selection-with-the-gamma-metric.

## Appendix A: Computation of the $\varvec{\gamma }$-metric

Let *S* be a set of *n* observations noted $\{ \varvec{X_i} \}_{i = 1,...,n}$, characterised by *p* features, where $\varvec{X_i} \in S \subset \mathbb {R}^p$. *S* is divided into *K* classes such that we have an integer vector $\varvec{Y}$ where $Y_{i} = 1,...,K \quad \forall i = 1,...,n$. For each $k \in \{1,...,K\}$, $\varvec{W}_{k,p}$ is the covariance matrix of the corresponding sub-sample of observations belonging to class *k*:

4 $$\begin{aligned} \varvec{W}_{k,p} = \text {Cov}(\varvec{X_{i}}|\varvec{Y_i} = k) \end{aligned}$$

where $\varvec{W}_{k,p}$ is a diagonizable $p \times p$ symmetrical positive semi-definite matrix in which all eigenvalues $\{ \lambda _{k, j}\}_{j = 1,...,p}$ are positive ($\forall j = 1,...,p, \quad \lambda _{k,j} \ge 0$). Let $\{ u_{k,j} \}_{j = 1,...,p}$ be the normalized eigenvectors associated with eigenvalues $\{ \lambda _{k,j} \}_{j = 1,...,p}$. These eigenvectors represent the directions of the *p* axes of length $\sqrt{\lambda _{k,j}}$ in a *p*-dimensional ellipsoid centred in $\varvec{\mu _k}$, which is the mean vector of observations in class *k*. Each class *k* (with $k \in \{1,...,K\}$) is thus represented by an ellipsoid in $\mathbb {R}^p$. Let $k_1, k_2 \in \{1,...,K\}$ such that $k_1 \ne k_2$ along the mean-mean axis given by $\varvec{\mu _{k_{1}}} \varvec{\mu _{k_{2}}} = \varvec{\mu _{k_{1}}} - \varvec{\mu _{k_{2}}}$ can be defined as follows:

$$\begin{aligned} d_{k_1, k_2} = \frac{1}{\alpha _{k_1, k_2}} \big (||\varvec{\mu _{k_1}} \varvec{\mu _{k_2}}|| - (d_{k_1, k_1 k_2} + d_{k_2, k_1 k_2}) \big ), \end{aligned}$$

where $\alpha _{k_1, k_2}$ is a normalisation factor defined as:

$$\begin{aligned} \alpha _{k_1, k_2} = \sqrt{\sum\limits_{j = 1}^p \lambda _{k_1, j}} + \sqrt{\sum\limits_{j = 1}^p \lambda _{k_2, j}}, \end{aligned}$$

and $d_{k_{1}, k_{1}k_{2}}$ and $d_{k_{2}, k_{1}k_{2}}$ are defined as :

$$\begin{aligned} d_{k_1, k_1k_2} = \frac{1}{\sqrt{\sum\nolimits_{j = 1}^{p} \frac{\tilde{\mu }^{2}_{k_{1}, j}}{\lambda _{k_1, j}}} } \quad \text {and} \quad d_{k_2, k_1k_2} = \frac{1}{\sqrt{\sum\nolimits_{j = 1}^{p} \frac{\tilde{\mu }^{2}_{k_{2}, j}}{\lambda _{k_2, j}}}}, \end{aligned}$$

where $\tilde{\mu }^{2}_{k_1, j}$ (respectively $\tilde{\mu }_{k_2,j}^{2}$) represents the coordinates of the normalised vector $\varvec{\mu }_{k_1}$, $\varvec{\mu }_{k_2}$ expressed in the orthogonal basis formed by the eigenvectors of ellipsoid $k_1$ (respectively $k_2$). If $\varvec{U}_{k_1}$ (respectively $\varvec{U}_{k_2}$) is the matrix whose columns correspond to the eigenvectors of ellipsoid $k_1$ (respectively $k_2$), then the normalized mean-mean vector $\varvec{\tilde{\mu }}_{k_1}$ (respectively $\varvec{\tilde{\mu }}_{k_2}$) can be written as:

$$\begin{aligned} \varvec{\tilde{\mu }}_{k_1} = \varvec{U}_{k_1}^{-1} \frac{\varvec{\mu }_{k_1} \varvec{\mu }_{k_2}}{||\varvec{\mu }_{k_1} \varvec{\mu }_{k_2}||_2} \quad \text {and} \quad \varvec{\tilde{\mu }}_{k_2} = \varvec{U}_{k_2}^{-1}\frac{\varvec{\mu }_{k_1} \varvec{\mu }_{k_2}}{||\varvec{\mu }_{k_1} \varvec{\mu }_{k_2}||_2}. \end{aligned}$$

$d_{k_1, k_1k_2}$ represents the distance between $\varvec{\mu }_{k_1}$ and the border of the ellipsoid. Any point on this border is determined by drawing a segment between $\varvec{\mu }_{k_1}$ and the border of the ellipsoid in the same direction as $\varvec{\mu }_{k_1} \varvec{\mu }_{k_2}$. Similarly, $d_{k_2, k_1k_2}$ is the distance between $\varvec{\mu }_{k_2}$ and the border of the ellipsoid, and any point on this border is determined by drawing a segment between $\varvec{\mu }_{k_2}$ and the border of the ellipsoid in the same direction as vector $-\varvec{\mu }_{k_1} \varvec{\mu }_{k_2}$. Finally, the $\gamma$-metric for a set of *K* classes of observations in $S \subset \mathbb {R}^p$ is defined as follows:

$$\begin{aligned} \gamma _p = \sum _{k_1 = 1}^{K} \sum _{k_1 < k_2} d_{k_1, k_2}. \end{aligned}$$

## Appendix B: Conventional feature selection methods’ computation

### Chi-squared

The Chi-squared statistic is computed for each discretized feature and class as follows:

$$\begin{aligned} \chi ^2 = \sum _{i = 1}^{2} \sum _{j=1}^{k} \frac{(A_{ij} - E_{ij})^2}{E_{ij}}, \end{aligned}$$

With *k* being the number of classes, $A_{ij}$ the number of observations in interval *i* and class *j*, and $E_{ij}$ the expected frequency of $A_{ij}$. Cramer’s V is used to obtain values between 0 and 1, with 1 being interpreted as a very strong correlation between the two features.

$$\begin{aligned} E_{ij} = \frac{R_{i} \times C_{j}}{N}, \end{aligned}$$

with $R_{i} = \sum _{j = 1}^{k} A_{ij}$ the number of observations in interval *i*, $C_{j} = \sum _{i = 1}^{2} A_{ij}$ the number of observations in class *j*, and $N = \sum _{i = 1}^{2} R_i$ the total number of observations.

### Correlation-based feature selection

To compute the importance score of the subsets we compute:

$$\begin{aligned} M = \frac{p_{S} \overline{r}_{cf}}{\sqrt{p_{S} + p_{S}(p_{S} - 1)\overline{r}_{ff}}}, \end{aligned}$$

with $p_S$ being the number of features in subset *S*, $\overline{r}_{cf}$ the average feature-class correlation, and $\overline{r}_{ff}$ is the average feature-feature correlation.

### Symmetrical uncertainty

Symmetrical uncertainty method is a variant of mutual information, or information gain, to lower the bias of the features with a large number of different values. To do so we operate a normalization of the mutual information. The mutual information measures the dependence between a feature $f_i$ and class *C* and being computed as follows:

$$\begin{aligned} \text {IG}(f_i, C) = H(f_i) - H(f_i|C), \end{aligned}$$

with $H(f_i)$ is the entropy of feature $f_i$ and $H(f_i|C)$ the conditional entropy of $f_i$ given *C*, which is defined as follows:

$$\begin{aligned} H(f_i) = - \sum _{x \in \text {supp}{(f_i)}} P(x)\log _{2}(P(x)), \end{aligned}$$

$$\begin{aligned} H(f_i|C) = - \sum _{k \in \{1,...,K \}} P(c_k) \sum _{x \in \text {supp}(f_i)} P(x|c_k) \log _2(P(x|c_k)), \end{aligned}$$

with *P*(*x*) being the probability that $f_i$ takes value *x*, $\text {supp}(f_i)$ the support of $f_i$, $P(c_k)$ the probability that *C* takes value $c_k$, and $P(x|c_k)$ the probability that $f_i$ takes the value *x* when *C* takes the value $c_k$. The symmetrical uncertainty is a normalize variant of $IG(f_i, C)$:

$$\begin{aligned} \text {SU}(f_i, C) = 2 \frac{\text {IG}(f_i, C)}{H(f_i) + H(C)}. \end{aligned}$$

## Appendix C: technique for order preference by similarity to ideal solution

The TOPSIS method can be implemented using the following procedure:

Step 1: Normalize the indicator values
  5 $$\begin{aligned} r_{ij} = \frac{x_{ij}}{\sqrt{\sum _{k = 1}^{J}x_{ik}^2}}, \end{aligned}$$
  with $x_{ij}$ represents the performance of feature selection method *j* on indicator *i*. The total number of methods is *J*, the total number of indicators is *m*.
Step 2: Calculate the weighted indicator values:
  6 $$\begin{aligned} v_{ij} = w_i r_{ij}, \end{aligned}$$
  where $w_i$ is the weight assigned to indicator *i*.
Step 3: Define the ideal and worst solutions
  Each indicator is classified as either benefit indicator (high values indicate better performance) or a cost indicator (lower values indicate better performance). The ideal solution $S^{+}$ maximizes all benefit indicators and minimizes all cost indicators, while the worst solution $S^{-}$ minimizes all benefit indicators and maximizes all cost indicators. The ideal and worst solutions can be defined as:
  7 $$\begin{aligned} S^{+} & = \big \{ \langle \max _j v_{ij} | i \in I^{\prime } \rangle , \langle \min _j v_{ij} | i \in I^{\prime \prime }\rangle \big \} = \big \{v^{+}_{i}|i \in \{1,\dots ,m\} \big \}\end{aligned}$$
  8 $$\begin{aligned} S^{-} & = \big \{ \langle \min _{j} v_{ij} | i \in I^{\prime } \rangle , \langle \max _{j} v_{ij} | i \in I^{\prime \prime } \rangle \big \} = \big \{ v^{-}_{i} | i \in \{1,\dots ,m\}\big \}, \end{aligned}$$
  where $I^{\prime }$ and $I^{\prime \prime }$ represent the sets of benefit and cost indicators, respectively.
Step 4: Compute the distances
  For each feature selection method *j*, we calculate the Euclidean distance to both the ideal solution $D_{j}^{+}$ and the worst solution $D_{j}^{-}$:
  9 $$\begin{aligned} D_{j}^{+}=\sqrt{\sum _{i = 1}^{m} (v_{ij} - v_{i}^{+})^2} \quad \text {and} \quad D_{j}^{-} \sqrt{\sum _{i = 1}^{m}(v_{ij} - v_{i}^{-})^2}. \end{aligned}$$
Step 5: Compute the relative closeness to the ideal solution
  The relative closeness $R_{j}^{+}$ to the ideal solution is calculated as:
  10 $$\begin{aligned} R_{j}^{+} = \frac{D_{j}^{-}}{D_{j}^{+} + D_{j}^{-}} \end{aligned}$$
  A high value of $R_{j}^{+}$ indicates that the method is closer to the ideal solution, with $R_{j}^{+} = 1$ representing the best possible performance and $R_{j}^{+} = 0$ representing the worst.
Step 6: Rank the feature selection methods
  The feature selection methods are ranked in decreasing order based on their $R_{j}^{+}$ values. The method with the highest $R_{j}^{+}$ offers the best compromise across all indicators.

To compute the $R_{j}^{+}$ values, we must first define the weights $w_{i}$ for each indicator. These weights reflect the emphasis placed on each indicator when assessing the performance of feature selection methods. Each weight $w_i$ lies within the range [0, 1] and must satisfy the condition $\sum _{i = 1}^{m} w_i = 1$.

## Appendix D: Justification of the choice of the $\beta$ values

In scenario 2, we generated classes based on two levels of balance (balanced/unbalanced) and separation (strong/weak), using three features with non-null effects and an intercept, $\beta _0$. As outlined in the data generation section, we computed a probability $\rho _i$ for each observation. Each observation’s class was determined through a Bernoulli process with parameter $\rho _i$. This section explains the choice of $\varvec{\beta } = (\beta _0, \beta _1, \beta _2, \beta _3)$ values according to the scenario we aimed to generate.

To recap, the probability $\rho _i$ for each observation $i \in \{1,\dots ,n\}$ is given by:

$$\begin{aligned} \rho _i = \frac{\exp (\varvec{X}_i \varvec{\beta })}{1+\exp (\varvec{X}_i \beta )} = \frac{1}{1 + \exp (-\varvec{X}_i \varvec{\beta })}. \end{aligned}$$

For simplicity, we focus on two features $X_1$ and $X_2$ with non-null effects. We express the equation of the decision boundary where $\rho _i = \frac{1}{2}$, which represents observations with a $50\%$ probability of being classified as 1. Thus $\forall i \in \{1,\dots ,n\}$, we have:

$$\begin{aligned} \frac{1}{1 + \exp \{-(\beta _0 + x_{i1}\beta _1 + x_{i2}\beta _2)\}} = \frac{1}{2} \end{aligned}$$

This simplifies to:

$$\begin{aligned} \forall \beta _2 \ne 0, x_2 = - \frac{\beta _0}{\beta _2} - \frac{\beta _1}{\beta _2}x_1, \end{aligned}$$

Observations near this line have an approximately $50\%$ probability of being in class 1. The values of $\varvec{\beta } = (\beta _0, \beta _1, \beta _2)$ determine the slope (i.e., $-\frac{\beta _1}{\beta _2}$) and intercept (i.e., $-\frac{\beta _0}{\beta _2}$) of this line, thereby influencing the distribution of the two classes.

In the case of a balanced dataset, where observations are centered around 0, the decision line should pass through the origin (0, 0). To achieve this, the intercept must be zero, i.e., $-\frac{\beta _0}{\beta _2} = 0$. By varying values of $\beta _0$, we control the degree of class imbalance.

For example, as shown in Fig. 4, when only the values of $\beta _0$ change (top panels of Fig. 4), the decision line shifts, reducing the balance between the two classes. Conversely, when $\beta _0 = 0$, altering $\beta _1$ and $\beta _2$ does not affect class balance, as all observations are drawn from a standard normal distribution.

For class separation, we used the Bernoulli distribution to generate random class labels based on the probability $\rho _i$ calculated for each observation. Rather than using a strict decision rule such as $Y_i = 1$ when $\rho _i \ge 0.5$, we allowed observations with $\rho _i \ge 0.5$ to still be classified as 0, introducing overlap between the classes. When the probabilities are concentrated around 0 or 1, the overlap is minimal, indicating strong separation.

To demonstrate this, we used the same matrix $\varvec{X}$ to generate the results shown in Fig. 5, which compares strong separation (left panel) and weak separation (right panel). In the strong separation case, the distribution of $\rho _i$ is concentrated near 0 and 1, while in the weak separation case, the distribution is flatter. In both scenarios, approximately 50% of the observations are labeled as 1. Thus, we control the degree of class overlap (i.e., separability) by adjusting the distribution of $\rho _i$.

## Appendix E: Visualisation of the correlation matrices and $\beta$ vector chosen in scenario 3 of the simulation study

In scenario 3, we generated data with multicollinearity by using a multivariate Gaussian distribution with a specific covariance matrix, $\varvec{\Sigma }$, which was structured as a block diagonal matrix. The following figures illustrate the matrix $\varvec{\Sigma }$ and the block matrix $\varvec{\Sigma }_g$ for a group of features under conditions of constant (Fig. 6) and non-constant (Fig. 7) correlation levels.

Additionally, we employed a specific $\varvec{\beta }$ vector with values of 1.5 or 0, distinguishing between informative and non-informative features. As shown in Fig. 8, each group contained ten features. In the first five groups, only the first feature was informative, while the rest were non-informative. Groups 6 through 9 consisted entirely of non-informative features, and group 10 was independent, with the first feature being informative and the others non-informative. As a result, only $\beta _{1}$, $\beta _{11}$, $\beta _{21}$, $\beta _{31}$, $\beta _{41}$ and $\beta _{91}$ were non-zero. The intercept term, $\beta _0$, was set to 0.
